# 

**Published:** 1897-01-23

**Authors:** 


					ime HutstiTAL, ABSOLUTELY PURE, therefore BEST, Jan. 23rd, 1897<
CADBURY'S tf CADBURY'S
COCOA V B JBT-
" The standard of highest purity at present M
flttftlnaltlik '1 ~ T rwAMf'/fl
attainable.*'?Za?cr*. <P> C?> ?-> NO ALKALIES USED.
GOOOA
ORIYICH HOSFl.TAL
?. ?^.WE TWOPE? Wil
No. 539. Vol. XXI. .1An! rot ?>? n i Iixmnr In K, aL. po?t*tr^'
aovmsTuwhirmMUX PBICE TWOPENCE.
Satubday,
Jan. 23rd, 1897.
[Registered at me wenerai r-osi umoe as a newspaper iot
transmission in the United Kingdom and Abroad.] Dtttoplr M?pSrt tee!

				

